# Sentinel Surveillance System Implementation and Evaluation for SARS-CoV-2 Genomic Data, Washington, USA, 2020–2021

**DOI:** 10.3201/eid2902.221482

**Published:** 2023-02

**Authors:** Hanna N. Oltean, Krisandra J. Allen, Lauren Frisbie, Stephanie M. Lunn, Laura Marcela Torres, Lillian Manahan, Ian Painter, Denny Russell, Avi Singh, JohnAric MoonDance Peterson, Kristin Grant, Cara Peter, Rebecca Cao, Katelynn Garcia, Drew Mackellar, Lisa Jones, Holly Halstead, Hannah Gray, Geoff Melly, Deborah Nickerson, Lea Starita, Chris Frazar, Alexander L. Greninger, Pavitra Roychoudhury, Patrick C. Mathias, Michael H. Kalnoski, Chao-Nan Ting, Marisa Lykken, Tana Rice, Daniel Gonzalez-Robles, David Bina, Kelly Johnson, Carmen L. Wiley, Shaun C. Magnuson, Christopher M. Parsons, Eugene D. Chapman, C. Alexander Valencia, Ryan R. Fortna, Gregory Wolgamot, James P. Hughes, Janet G. Baseman, Trevor Bedford, Scott Lindquist

**Affiliations:** Washington State Department of Health, Shoreline, Washington, USA (H.N. Oltean, K.J. Allen, L. Frisbie, S.M. Lunn, L.M. Torres, L. Manahan, I. Painter, D. Russell, A. Singh, J.M. Peterson, K. Grant, C. Peter, R. Cao, K. Garcia, D. Mackellar, L. Jones, H. Halstead, H. Gray, G. Melly, S. Lindquist);; University of Washington, Seattle, Washington (H.N. Oltean, D. Nickerson, L. Starita, C. Frazar, A.L. Greninger, P. Roychoudhury, P.C. Mathias, J.P. Hughes, J.G. Baseman);; Northwest Genome Center, Seattle (D. Nickerson, L. Starita, C. Frazar);; Atlas Genomics, Seattle (M.H. Kalnoski, C.-N. Ting);; Confluence Health, Wenatchee, Washington (M. Lykken, T. Rice, D. Gonzalez-Robles);; Incyte Diagnostics, Spokane Valley, Washington (D. Bina, K. Johnson, C.L. Wiley);; Interpath Laboratory, Boise, Idaho, USA (S.C. Magnuson, C.M. Parsons, E.D. Chapman, A. Valencia);; Northwest Laboratory, Bellingham, Washington (R.R. Fortna, G. Wolgamot);; Fred Hutchinson Cancer Research Center, Seattle (T. Bedford)

**Keywords:** COVID-19, respiratory infections, severe acute respiratory syndrome coronavirus 2, SARS-CoV-2, SARS, coronavirus disease, zoonoses, viruses, coronavirus, epidemiology, surveillance, genomics, Washington, United States

## Abstract

Genomic data provides useful information for public health practice, particularly when combined with epidemiologic data. However, sampling bias is a concern because inferences from nonrandom data can be misleading. In March 2021, the Washington State Department of Health, USA, partnered with submitting and sequencing laboratories to establish sentinel surveillance for SARS-CoV-2 genomic data. We analyzed available genomic and epidemiologic data during presentinel and sentinel periods to assess representativeness and timeliness of availability. Genomic data during the presentinel period was largely unrepresentative of all COVID-19 cases. Data available during the sentinel period improved representativeness for age, death from COVID-19, outbreak association, long-term care facility–affiliated status, and geographic coverage; timeliness of data availability and captured viral diversity also improved. Hospitalized cases were underrepresented, indicating a need to increase inpatient sampling. Our analysis emphasizes the need to understand and quantify sampling bias in phylogenetic studies and continue evaluation and improvement of public health surveillance systems.

Virus genome data can provide useful information for public health practice, particularly when combined with epidemiologic data in real time. Goals of genomic surveillance can include monitoring circulating and emerging variants, detecting and characterizing outbreaks, describing spatiotemporal patterns of virus transmission, supporting epidemiologic and genomic characterization of variants, and pinpointing introduction sources that might be risk factors (*1*). Information from a paired genomic and epidemiologic surveillance system can then be translated into public health interventions to prevent disease, control spread, and mitigate outbreaks. Interventions could include planning preparedness according to emerging variant characteristics, changing therapeutic and nonpharmaceutical interventions, and recommending control strategies on the basis of outbreak characteristics. To ensure generalizability and equity when using paired genomic and epidemiologic data for public health purposes, the methods for capturing those data must ensure a representative sample from the population of interest (*2*,*3*).

Ongoing global circulation of SARS-CoV-2 and repeated emergence of new variants indicate the need for robust genomic surveillance to inform public health responses (*4*). In Washington, USA, surveillance of SARS-CoV-2 is passive and, therefore, focused on cases of COVID-19 in persons seeking testing. In addition, methods for conducting next-generation sequencing introduce limitations on sampling; specimens must contain adequate quantities of viral RNA for sequencing efforts to be successful. Therefore, persons who had mild illness, delayed testing, reinfection, or other characteristics that might lower viral loads are less likely to be represented in sequencing data. Knowing those limitations, the Washington State Department of Health sought to establish a genomic sentinel surveillance system for SARS-CoV-2 in March 2021.

Before sentinel surveillance was initiated, large amounts of genomic data were produced by academic and clinical laboratories in Washington and shared publicly via the GISAID EpiCoV database (*5*–*7*). Studies using those data to rapidly produce critical viral transmission and evolution information were published early during the pandemic; however, the populations captured in those data remain unknown (*8*–*12*). Sampling bias or systematic differences in sample characteristics between COVID-19 cases with sequenced specimens and total COVID-19 cases is a concern. Using large datasets from a limited number of geographically sparse institutions might produce inaccurate phylogenetic representations of virus distribution and migration within the population (*13*,*14*). Specifically, discrete trait analysis is a type of phylogeographic analysis that treats lineage migration between locations as if the location was a discrete trait; models relying on this analysis type assume that sample sizes across subpopulations are proportional to their relative size and random sampling occurs (*15*). If 1 population is oversampled, large biases are expected in model output (*15*). This concern extends beyond state or country borders because representative sampling is often assumed for contextual data, which provides the backdrop upon which phylogenetic inference is based.

We describe implementing a sentinel surveillance system that enables pairing of genomic and epidemiologic data. In addition, we assessed representativeness and timeliness of genomic data availability before and after system implementation. By performing this evaluation, we provide information regarding populations of sampled cases and limitations on inference affecting genomic data use. To support planning efforts to obtain more equitable and representative sampling, we identified subpopulations that might be systematically excluded from sequencing surveillance. More broadly, we raise awareness regarding sampling bias in convenience-based genomic surveillance systems and support development of robust genomic surveillance systems in additional jurisdictions.

## Methods

### Sentinel Surveillance System Design

In March 2021, the Washington State Department of Health partnered with multiple laboratories to establish a sentinel surveillance program to monitor genomic epidemiology of SARS-CoV-2 within the state. Partner laboratories were selected to maximize geographic coverage and specimen numbers. The initial proportion of randomly selected positive specimens submitted for sequencing was designed to balance geographic coverage regionally and match available sequencing capacity; statewide case coverage varied from 8% to 25% during the study period (*16*). In addition to the Washington State Public Health Laboratories, the 6 sentinel laboratories are Atlas Genomics, Confluence Health/Central Washington Hospital, Interpath Laboratories, Incyte Diagnostics Spokane, Northwest Laboratories, and University of Washington Virology Division. PCR cycle threshold (Ct) is capped at 30 for this surveillance system. The surveillance program is supplemented by a national surveillance effort supported by the Centers for Disease Control and Prevention (CDC), which includes multiple commercial laboratories sequencing randomly selected specimens (*2*). Methods for next-generation sequencing vary across laboratories, but >90% sequences are generated by using an Illumina platform (https://www/illumina.com); assembly methods also vary.

### Study Population Evaluation

We included all confirmed COVID-19 cases (SARS-CoV-2 RNA detected by molecular amplification) reported in the Washington Disease Reporting System from January 21, 2020, through December 31, 2021. Using laboratory accession numbers or patient demographics, we linked those cases to sequences uploaded to the GISAID EpiCoV database (*5*–*7*) from January 21, 2020, through January 31, 2022, that indicated the state of Washington in the geographic tag. We classified cases as presentinel surveillance if specimens were sequenced before March 1, 2021. We classified cases as sentinel surveillance if specimens were sequenced on or after March 1, 2021, and submitted through the Washington State Department of Health sentinel surveillance program, or if the sequencing laboratory indicated that specimens were randomly selected. Specimens specifically selected for targeted sequencing as part of outbreak investigations because of travel history, known vaccine breakthrough status, or spike gene target failures were not considered sentinel surveillance if sampled outside the random selection process. Washington state and University of Washington Institutional Review Boards determined this project to be a surveillance activity and exempt from review.

### Data Analysis

We assessed representativeness of data before and after implementing sentinel surveillance by comparing COVID-19 cases with sequenced specimens to all COVID-19 cases during the same period according to sex, age, race, ethnicity, language, long-term care facility (LTCF) association, occupation, county of residence, outbreak association, travel history, hospitalization, or death. All epidemiologic data analyses were performed using R version 4.0.3 (*17*). We compared categorical data by using Pearson χ^2^ test or the formula Σ(|E-O|)/E, where E was expected and O observed counts. Expected counts were calculated by standardization to overall reported cases during the same period. We visualized geographic comparisons by mapping standardized ratios of observed versus expected cases at the county level. We graphed the percentage of cases with sequenced specimens by county and month to visualize spatiotemporal sampling. We evaluated areas with high presentinel sequencing coverage and high or low sentinel sequencing coverage to determine representativeness because data from those areas enabled robust phylogeographic studies.

To determine variability of genomic data, we constructed phylogenetic trees for 4 scenarios using the Nextstrain (*18*) pipeline for SARS-CoV-2. The scenarios were presentinel surveillance with high coverage, low representativeness; presentinel surveillance with high coverage, high representativeness; sentinel surveillance with high coverage, high representativeness; and sentinel surveillance with low coverage, low representativeness. We performed rarefaction analysis to examine how sampling affected the diversity of sequences captured in each of those 4 scenarios. For each value from 1 to n, where n is the total number of available sequences for a location/timeframe of interest, we generated 10 subsampled datasets (sampling without replacement). We counted and plotted the number of unique haplotypes as a function of the number of sampled sequences.

We assessed timeliness of data by comparing the interval between initial specimen collection and genomic data upload to the GISAID database. We assessed median timeliness by month and compared categorical data uploaded within <14 days, 14–27 days, and >28 days after specimen collection.

## Results

During the presentinel surveillance period, 10,653 (3.3%) COVID-19 cases had sequencing information available, compared with 56,106 (12.1%) cases sampled during sentinel surveillance. For all categorical comparisons using Pearson χ^2^ tests, we observed statistically significant differences between presentinel and sentinel cases that had sequencing data. To avoid having a single large discrepancy dominate the representativeness measurement, we used the formula Σ(|E-O|)/E instead of Pearson χ^2^ test to directly compare representativeness between populations (Table).

**Table:** Comparison of demographic characteristics between COVID-19 cases with sequenced specimens and all confirmed COVID-19 cases in study of presentinel and sentinel surveillance system implementation and evaluation for SARS-CoV-2 genomic data, Washington, USA, 2020–2021*

Variable	Presentinel period†					Sentinel period‡			
	Overall	Sequenced	O/E	Σ(|E-O|)/E§		Overall	Sequenced	O/E	Σ(|E-O|)/E§
Total no.	326,850	10,653				463,639	56,106		
Sex				0.73					0.55
F	159,460 (48.8)	5,326 (50.0)	1.02			230,524 (49.7)	27,163 (48.4)	0.97	
M	157,133 (48.1)	4,932 (46.3)	0.96			223,711 (48.3)	27,916 (49.8)	1.03	
Other	287 (0.1)	NA¶	0.53			331 (0.1)	55 (0.1)	1.37	
Missing	9,970 (3.1)	390 (3.7)	1.20			9,073 (2.0)	972 (1.7)	0.89	
Age group, y				2.36					1.58
0–4	7,802 (2.4)	211 (2.0)	0.83			18,499 (4.0)	2,188 (3.9)	0.98	
5–17	32,121 (9.8)	932 (8.7)	0.89			77,782 (16.8)	9,815 (17.5)	1.04	
18–44	165,920 (50.8)	5,128 (48.1)	0.95			224,380 (48.4)	28,909 (51.5)	1.06	
45–64	83,046 (25.4)	2,628 (24.7)	0.97			102,215 (22.0)	11,309 (20.2)	0.91	
65–79	26,724 (8.2)	1,073 (10.1)	1.23			32,000 (6.9)	3,052 (5.4)	0.79	
>80	10,998 (3.4)	680 (6.4)	1.90			8,591 (1.9)	832 (1.5)	0.80	
Unknown	239 (0.1)	NA¶	0.13			172 (0.0)	NA¶	0.05	
COVID-19 deaths	5,134 (1.6)	448 (4.2)	2.68	1.68		4,568 (1.0)	452 (0.8)	0.82	0.18
Hospitalized for COVID-19	18,992 (5.8)	891 (8.4)	1.44	0.44		25,060 (5.4)	1,721 (3.1)	0.57	0.43
Outbreak-associated	49,165 (15.0)	2,350 (22.1)	1.47	0.47		25,902 (5.6)	4,281 (7.6)	1.37	0.37
LTCF-associated	19,899 (6.1)	1,614 (15.2)	2.49	1.49		7,317 (1.6)	1,105 (2.0)	1.25	0.25
Symptoms				0.61					0.80
Yes	172,070 (52.6)	6,860 (64.4)	1.22			173,363 (37.4)	27,140 (48.4)	1.29	
No	24,182 (7.4)	701 (6.6)	0.89			44,731 (9.6)	3,430 (6.1)	0.63	
Unknown	130,598 (40.0)	3,092 (29.0)	0.73			245,545 (53.0)	25,536 (45.5)	0.86	
Race/Ethnicity				1.95					1.35
Hispanic	70,020 (21.4)	2,671 (25.1)	1.17			53,221 (11.5)	9,285 (16.5)	1.44	
Non-Hispanic, American Indian, or Alaska Native	3,953 (1.2)	161 (1.5)	1.25			5,455 (1.2)	685 (1.2)	1.04	
Non-Hispanic Asian	16,321 (5.0)	755 (7.1)	1.42			21,787 (4.7)	3,261 (5.8)	1.24	
Non-Hispanic Black	14,863 (4.5)	548 (5.1)	1.13			19,812 (4.3)	2,429 (4.3)	1.01	
Non-Hispanic multiracial	5,575 (1.7)	217 (2.0)	1.19			7,707 (1.7)	1,173 (2.1)	1.26	
Non-Hispanic Native Hawaiian or other Pacific Islander	5,338 (1.6)	203 (1.9)	1.17			6,432 (1.4)	704 (1.3)	0.90	
Non-Hispanic White	133,224 (40.8)	4,174 (39.2)	0.96			229,100 (49.4)	24,039 (42.8)	0.87	
Non-Hispanic, other race	3,211 (1.0)	138 (1.3)	1.32			3,271 (0.7)	345 (0.6)	0.87	
Unknown	74,345 (22.7)	1,786 (16.8)	0.74			116,854 (25.2)	14,185 (25.3)	1.00	
Language				0.94					2.15
English	104,984 (32.1)	3,357 (31.5)	0.98			138,437 (29.9)	18,484 (32.9)	1.10	
Spanish	23,408 (7.2)	884 (8.3)	1.16			9,849 (2.1)	2,474 (4.4)	2.08	
Other	5,137 (1.6)	239 (2.2)	1.43			1,745 (0.4)	337 (0.6)	1.60	
Unknown	12,519 (3.8)	273 (2.6)	0.67			9,261 (2.0)	1,434 (2.6)	1.28	
Missing	180,802 (55.3)	5,900 (55.4)	1.00			304,347 (65.6)	33,377 (59.5)	0.91	

*Values are no. or no. (%). We included all confirmed COVID-19 cases (SARS-CoV-2 RNA detected by molecular amplification) reported among Washington residents from January 21, 2020, through December 31, 2021, in the Washington Disease Reporting System. E, expected counts; LTCF, long-term care facility; NA, not applicable; O, observed counts. †Cases were classified as presentinel if specimens were sequenced before March 1, 2021. ‡Cases were classified as sentinel if specimens were sequenced on or after March 1, 2021, through the sentinel surveillance program. §Formula used to directly compare representativeness between populations. ¶Counts <10 are censored.

Both presentinel and sentinel cases with sequencing data were generally representative of all COVID-19 cases for sex at birth. During the presentinel surveillance period, older age groups and hospitalized persons with sequenced specimens were overrepresented. Persons who died of COVID-19 were overrepresented by ≈3-fold among presentinel cases with sequencing data compared with cases that had no sequencing data. Sentinel surveillance implementation resolved overrepresentation of decedents, but persons with COVID-19 who were hospitalized or >65 years of age were underrepresented.

Early during the pandemic, specimens from known outbreak-associated COVID-19 cases were more commonly sequenced, likely reflecting preferential sample selection of those cases for studies. Similarly, sequencing of specimens from LTCF-associated COVID-19 cases was enriched by 2.5-fold. Sentinel surveillance implementation decreased but did not completely resolve enrichment of outbreak-associated cases, whereas LTCF-associated case enrichment was substantially resolved.

Presentinel COVID-19 cases with sequenced specimens had more complete symptom information when compared with all COVID-19 cases. Both presentinel and sentinel cases with sequenced specimens had symptom information reported more frequently compared with all cases. 

Persons self-reporting as a racial or ethnic minority were generally overrepresented among presentinel COVID-19 cases with sequenced specimens; race/ethnicity data were less likely to be missing among those cases than among total COVID-19 cases. After sentinel surveillance implementation, persons reporting Hispanic ethnicity or Spanish language preference were overrepresented among COVID-19 cases with sequenced specimens. Differences in missing race data were resolved after sentinel surveillance implementation.

Industry information was missing for most cases. According to the available industry information, agriculture, forestry, fishing and hunting, and healthcare and social assistance were overrepresented among cases with sequenced specimens. Industry information was missing for >90% of cases during the sentinel surveillance period; therefore, industry representation was not assessed in this study.

More persons with sequenced specimens during the presentinel period traveled outside the United States than expected, indicating likely enrichment for international travelers. Travel information was missing for >95% of cases during the sentinel surveillance period; therefore, traveler representation was not assessed in this study.

Reinfection data were captured starting on September 1, 2021; therefore, case-level data were not available for most of the study period. From September through December 2021, reinfection cases were underrepresented in the sequencing data, which might reflect a higher average Ct in this population.

**Figure 1:**
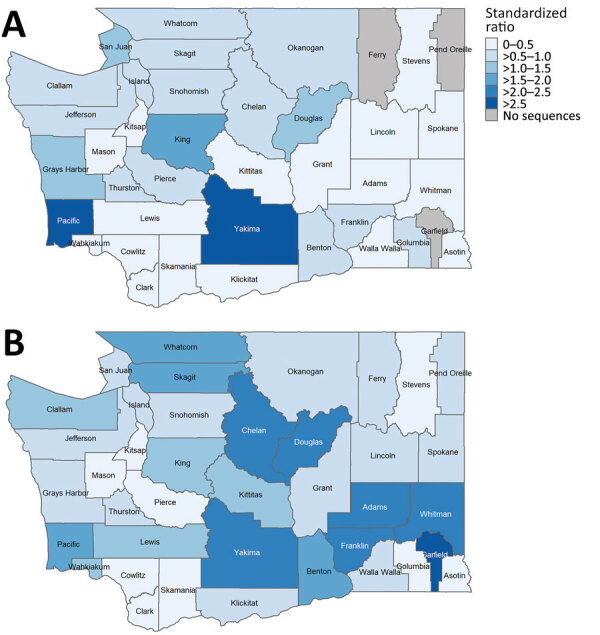
Geographic extent of sequencing data available for COVID-19 cases in study of sentinel surveillance system implementation and evaluation for SARS-CoV-2 genomic data, Washington, USA, 2020–2021. A) Presentinel surveillance (specimens sequenced before March 1, 2021). B) Sentinel surveillance (specimens sequenced on or after March 1, 2021, through the sentinel surveillance program). Standardized ratios (observed/expected counts) of cases with sequenced specimens are indicated by county. No sequence data were available for 3 counties during the presentinel period.

Before sentinel surveillance implementation, geographic sequencing coverage was variable and focused on western Washington (Figure 1); King, San Juan, Pacific, and Yakima Counties had high coverage. Some areas of the state had little or no data available. After sentinel surveillance implementation, geographic coverage equalized regionally across the state; variable coverage because of sentinel laboratory service areas occurred as expected (Figure 1).

We investigated representativeness further in areas with high presentinel sequencing coverage and high cases numbers (Appendix Figure 1). During March–June 2020, Yakima County had 19%–30% sequencing coverage for all COVID-19 cases; high-quality genomic data were available for 1,696 cases. High coverage was partially driven by sequencing specimens from LTCF-associated cases. A total of 25% of cases with sequenced specimens were affiliated with LTCFs, compared with 11% of all COVID-19 cases during that period. Persons with sequenced specimens were more commonly >65 years of age and less commonly of Hispanic descent or with Spanish language preference.

We performed phylogenetic analysis of all sequenced specimens from Yakima County cases with COVID-19 onset dates during March–June 2020 (Appendix Figure 2, panel A). During this period, most (63%) sequences were classified as Nextstrain clade 20B (Pango lineage B.1.1), 23% were clade 19B (Pango lineage A), 9% were clade 20A (Pango lineage B.1) and 5% were clade 20C. Comparatively, within the entire state of Washington, clades 20C and 19B (Pango lineage A) were most prevalent during the same period.

Sequencing coverage was also high in Yakima County in February 2021. Sequencing coverage was 26% across all COVID-19 cases, and high-quality genomic data were available for 271 cases. During this period, we observed smaller differences between cases with sequenced specimens and all cases for ethnicity and outbreak-association; otherwise, cases with sequenced specimens were largely representative of all cases during this time. We performed phylogenetic analysis of Yakima cases during February 2021 (Appendix Figure 2, panel B). The most common lineage identified was 21C (Pango lineage B.1.427/429 or Epsilon), representing 33% of sequences, then 20G (Pango lineage B.1.2) at 29%, 20A at 13%, 20B at 9%, and 20C at 15%. In Washington, 30% of sequences in GISAID were Epsilon in February 2021.

After sentinel surveillance implementation, variability in geographic coverage was diminished regionally but persisted at the county level. We investigated counties with high and low sentinel sequencing coverage to determine effects of variable sentinel specimen sampling. We specifically compared Whatcom County, a county with high coverage from a sentinel laboratory, and Clark County, a county with low coverage. During the sentinel surveillance period, cases with sequenced specimens from Whatcom County were representative of all COVID-19 cases from the county for age, sex, race, death from COVID-19, and LTCF-association. Persons hospitalized for COVID-19 were underrepresented among sentinel surveillance cases, reflecting statewide findings. Outbreak-associated cases and symptomatic persons were slightly overrepresented among sentinel surveillance cases. We performed phylogenetic analysis of cases from Whatcom County during the sentinel surveillance period (Appendix Figure 2, panel C) and showed a transition from clade 20I (Alpha) to 21A/21I/21J (Delta) dominance, similar to what was observed in Washington overall.

**Figure 2:**
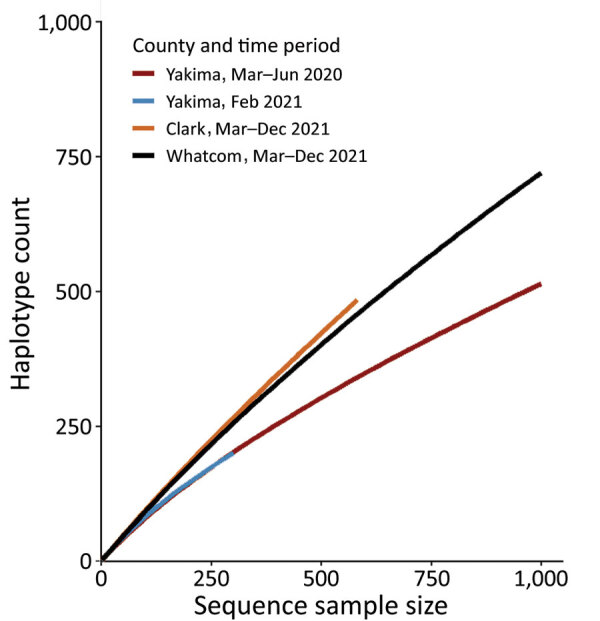
Rarefaction analysis of virus haplotype diversity in Yakima, Clark, and Whatcom Counties in study of sentinel surveillance system implementation and evaluation for SARS-CoV-2 genomic data, Washington, USA, 2020–2021. Presentinel COVID-19 cases (sequenced before March 1, 2021) with sequenced specimens from Yakima County (2 timepoints) were compared with sentinel COVID-19 cases (sequenced on or after March 1, 2021, through the sentinel surveillance program) with sequenced specimens in Clark and Whatcom Counties. Haplotype count indicates virus diversity.

Clark County had very low sequencing coverage over the sentinel surveillance period, ranging from 0.8% of cases in April 2021 to 4.9% of cases in June 2021. Persons <45 years of age and outbreak-associated cases were overrepresented among cases with sequenced specimens, and hospitalized persons were underrepresented. We performed phylogenetic analysis of cases from Clark County during the sentinel surveillance period (Appendix Figure 2, panel D). Despite limited coverage, we observed a variant profile similar to that of Whatcom County and Washington overall. We performed rarefaction analysis and found sentinel sampling from Clark and Whatcom counties displayed higher viral diversity than Yakima County at 2 presentinel timepoints (Figure 2). Additional sampling will be required in all scenarios to fully capture circulating viral diversity.

**Figure 3:**
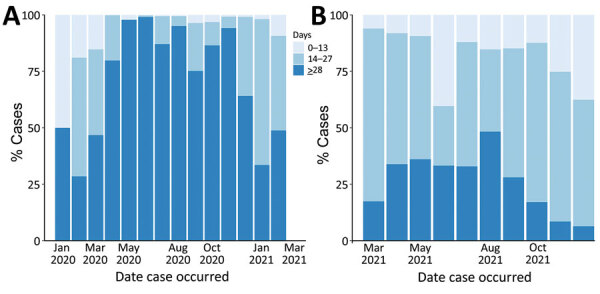
Timeliness of sequence data availability in study of sentinel surveillance system implementation and evaluation for SARS-CoV-2 genomic data, Washington, USA, 2020–2021. Graph shows percentages of COVID-19 cases with sequenced data uploaded to the GISAID database (https://www.gisaid.org) within 0–13, 14–27, and >28 days after specimen collection. A) Presentinel surveillance (specimens sequenced before March 1, 2021). B) Sentinel surveillance (specimens sequenced on or after March 1, 2021, through the sentinel surveillance program).

Timeliness of available genomic data in the GISAID database varied over the study period (Figure 3). During the presentinel period, median timeliness ranged from 23 days in February to 98 days in October of 2020; >50% of sequences were uploaded to GISAID >28 days after specimen collection for most months. During the sentinel period, median timeliness was 26 days in August and 15 days in December of 2021; most sequences were uploaded to GISAID <28 days after specimen collection in all months after sentinel surveillance implementation.

## Discussion

After a sentinel surveillance system for sequencing SARS-CoV-2 specimens was implemented in Washington, the available data were more epidemiologically and genomically representative of all COVID-19 cases and timelier than data before sentinel surveillance began. Specifically, representativeness of age, death from COVID-19, outbreak-association status, LTCF-affiliated status, and geographic coverage improved; increased viral diversity was also noted. Before sentinel surveillance began, we were unable to identify a county or period with representative sampling, except for Yakima County during February 2021. After implementation, representativeness improved across multiple areas. Increased representativeness is a critical achievement because genomic data are routinely available to public health leaders and decision-makers; ensuring equitable sampling coverage has substantial implications for response planning and interventions. Measuring effects of genomic surveillance on public health responses in Washington was not included in this study; however, methods for measuring and evaluating effectiveness should be explored.

Overrepresentation of older persons in presentinel genomic data was partly driven by selection of LTCF-associated COVID-19 cases and COVID-19 cases resulting in hospitalization or death. After sentinel surveillance began, the decrease in representation of persons >65 years of age improved overall representativeness but actually resulted in undersampling this age group, possibly indicating poor sequencing coverage by facilities where this population seeks care. Indeed, the sentinel surveillance system underrepresents hospitalized cases; further consideration is needed to improve data capture of both inpatient and outpatient COVID-19 cases. Before sentinel surveillance, outbreak-associated and symptomatic COVID-19 cases were oversampled. After implementation, overrepresentation of those cases decreased but was not resolved. At least 3 possible explanations exist for those findings: specimens from symptomatic SARS-CoV-2–infected persons are more likely to be sequenced because of higher average viral loads, which improves sequencing success; asymptomatic persons might be detected through screening programs not associated with sentinel laboratories; and outbreak-associated specimens might be sent to sentinel laboratories to ensure sequencing for investigative purposes. Random sampling among specimens received at sentinel laboratories could, thereby, still lead to biased samples.

Minority race and ethnicity were more commonly reported among presentinel cases with sequenced specimens; data were also more complete among those cases. Whether true overrepresentation occurred or race data were differentially missing among all cases is unclear. After sentinel surveillance implementation, persons reporting Hispanic ethnicity and Spanish language preference were overrepresented compared with overall cases statewide, which likely reflects the catchment areas of sentinel laboratories. Geographic coverage variability was identified during both presentinel and sentinel surveillance periods. Presentinel coverage focused on western Washington, where laboratories were connected to sequencing capacity. Sentinel surveillance enabled access to sequencing for additional laboratories and ensured greater equitable regional coverage, although variability at the county and subcounty levels remains. Variable coverage and representativeness at the substatewide level should be considered when using genomic data for specific analyses. Increasing geographic coverage will require additional sentinel laboratories that contribute specimens from areas of low coverage.

Other epidemiologic information was of interest in assessing representativeness, including industry and occupation, travel history, and reinfection status. However, data for those variables was incomplete, limiting their usefulness. As public health systems pivot away from capturing data through individual case interviews, datasets available for assessing sampling of specimens for sequencing should be considered. The full potential of genomic epidemiologic surveillance for improving public health requires pairing epidemiologic metadata with genomic data.

Viral diversity has been and continues to be dynamic over the course of the COVID-19 pandemic. Measuring true viral diversity requires random or complete sampling. Actual circulating viral diversity likely differed across locations and timepoints included in our study; if circulating diversity generally increased over time, our conclusions would be biased toward assumption of improved capture because of surveillance.

Other states and countries have used various practices to select SARS-CoV-2 specimens for sequencing. Methods that rely on convenience samples, such as our presentinel system, likely have sampling biases that affect phylogenetic inference. In those settings, weighting cases for inclusion in estimates by using selection probabilities might help to correct bias. Alternatively, approaches to correct for nonrepresentative sampling during analysis, such as inverse probability weighting, should be considered. Even after sentinel surveillance system is put in place, some biases remain, such as undersampling of hospitalized cases, that should be corrected by diversifying sources of specimens. Ongoing evaluation and improvement of systems is necessary, especially in the context of performing epidemiologic studies. Many epidemiologic studies of COVID-19 have availability of genomic data as an inclusion criterion; if sampling biases are not clarified, biased conclusions might be drawn. Co-development of genomic epidemiology programs alongside bioinformatics programs is needed in public health departments because epidemiologic and phylogenetic analyses are best performed after sampling methods and data limitations are considered.

Although representativeness and timeliness were the focus of this study, other features should be considered in the design of surveillance systems, such as simplicity, flexibility, sensitivity, and stability (*4*). Sentinel surveillance systems are complicated and require ongoing coordination with laboratory partners; stability requires public health resources. Alternative systems to enable representativeness and timeliness while increasing simplicity and stability could include requirements for specimen submission, such as those commonly used for foodborne pathogens and other notifiable conditions. Sensitivity is essential for the surveillance system goals of rare variant detection and timely surveillance of circulating virus variants. Right-size sampling, such as that performed for influenza surveillance, should be considered (*19*; S. Wohl et al., unpub. data, https://www.medrxiv.org/content/10.1101/2021.12.30.21268453v1).

Even after careful consideration of surveillance system design for pathogen sequencing and pairing with epidemiologic data, limitations remain because of specimen requirements for sequencing. Studies using surveillance sequencing data should report the following limitations: application of laboratory-based diagnostic testing might depend on many factors that are difficult to assess and increasingly complex because of availability of improved at-home testing, and, among positive test results, those with a low PCR Ct are more likely to be sequenced. Therefore, representativeness of sequencing data is inherently limited.

Assessment of representativeness during presentinel and sentinel surveillance is limited in the causal inferences that can be drawn. Other concurrent factors might have affected representativeness and timeliness during this study period. For example, CDC surveillance efforts were also increased during this timeframe; samples sequenced under CDC surveillance were coded as sentinel and were analyzed as part of the sentinel surveillance system in Washington.

In conclusion, implementing a sentinel surveillance system for sequencing SARS-CoV-2 specimens was associated with improved genomic and epidemiologic representativeness and timeliness of available sequence data in Washington. Ongoing evaluation and improvements will be necessary to ensure representative capture of inpatient settings. As public health leaders discuss changes to COVID-19 surveillance systems nationally, datasets required to assess representativeness of sampling for sequencing should be considered. Cross-jurisdictional sampling bias is a concern when validating phylogeographic methods applications; attention to sampling will improve the usefulness of those datasets for public health practice.

Appendix Additional information for sentinel surveillance system implementation and evaluation for SARS-CoV-2 genomic data, Washington, USA, 2020–2021.
