# Supplementary material for: Sentinel Surveillance System Implementation and Evaluation for SARS-CoV-2 Genomic Data, Washington, USA, 2020–2021
Source: Emerg Infect Dis. 2023 Feb;29(2):242–51. doi: 10.3201/eid2902.221482 (PMC9881772; doi:10.3201/eid2902.221482)
Supplement: Appendix — Additional information for sentinel surveillance system implementation and evaluation for SARS-CoV-2 genomic data, Washington, USA, 2020–2021. [file 22-1482-Techapp-s1.pdf]

Article DOI: https://doi.org/10.3201/eid2902.221482

Sentinel Surveillance System Implementation and Evaluation for SARS-CoV-2 Genomic Data, Washington, USA, 2020–2021

Appendix

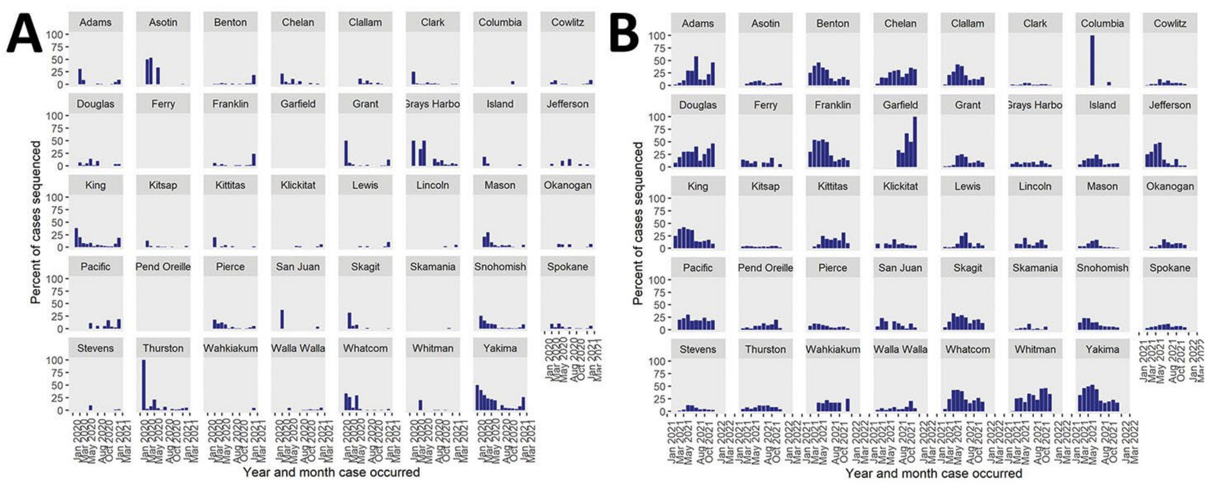

Appendix Figure 1. Percentages of COVID-19 cases with sequenced specimens by county, month, and year in study of sentinel surveillance system implementation and evaluation for SARS-CoV-2 genomic data, Washington, USA, 2020–2021. Presentinel specimens were sequenced before March 1, 2021; sentinel specimens were sequenced on or after March 1, 2021, through the sentinel surveillance program. A) Presentinel surveillance. B) Sentinel surveillance.

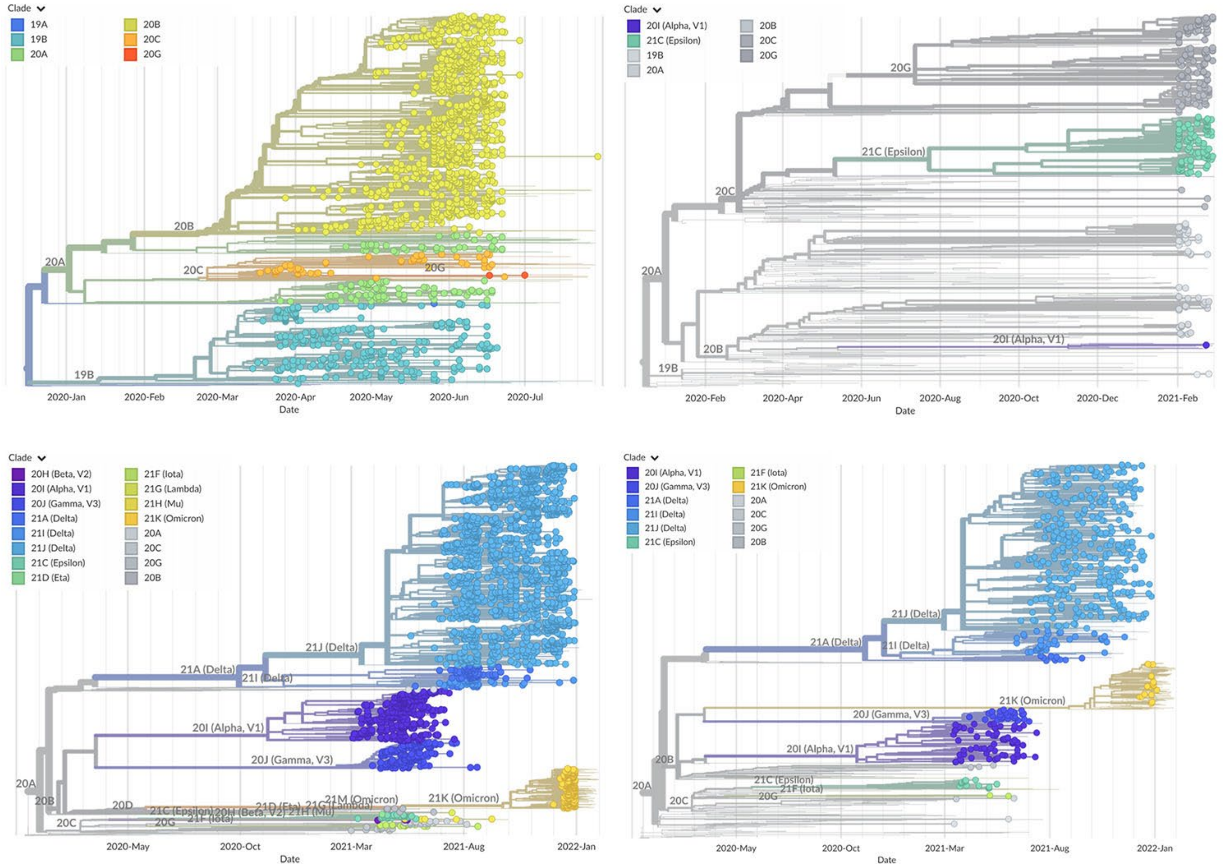

Appendix Figure 2. Time-scaled phylogenetic analysis of sequence data from Yakima, Clark, and Whatcom Counties in study of sentinel surveillance system implementation and evaluation for SARS-CoV-2 genomic data, Washington, USA, 2020–2021. Trees show SARS-CoV-2 specimens from presentinel COVID-19 cases in Yakima County (2 timepoints) and from sentinel COVID-19 cases in Clark and Whatcom Counties. Presentinel specimens were sequenced before March 1, 2021; sentinel specimens were sequenced on or after March 1, 2021, through the sentinel surveillance program. A) Yakima County, March–June 2020. B) Yakima County, February 2021. C) Whatcom County, March–December 2021. D) Clark County, March–December 2021.
